# Varicella Zoster Virus Encephalitis

**DOI:** 10.5811/cpcem.2019.8.43010

**Published:** 2019-10-14

**Authors:** Jared Lizzi, Tyler Hill, Julian Jakubowski

**Affiliations:** Memorial Health System, Department of Emergency Medicine, Marietta, Ohio

## Abstract

Varicella zoster virus in the adult patient most commonly presents as shingles. Shingles is a painful vesicular eruption localized to a specific dermatome of the body. One of the potential complications of this infection is involvement of the central nervous system causing encephalitis. An increased risk of this complication is associated with the immunocompromised patient. In this case report, we review the history and physical exam findings that should raise clinical suspicion for varicella zoster encephalitis, as well as the epidemiology, risk factors, treatment, and prognosis of this type of infection.

## INTRODUCTION

We present a case of a patient with varicella zoster virus (VZV) encephalitis caused by a combination of the patient having active virus reactivation in the form of shingles on the right leg, in addition to being immunocompromised due to a kidney transplant. According to the World Health Organization, encephalitis occurs in one out of every 33,000–50,000 cases of VZV. It also carries a less favorable prognosis compared to the other extracutaneous complications of VZV. This case report shows how prompt recognition and treatment of this type of infection can decrease mortality and progression of the infection in the high-risk, immunocompromised patient.

## CASE REPORT

A 67-year-old man with a medical history of kidney transplant, chronic renal dysfunction, prior cytomegalovirus infection causing retinal damage and vision loss and prescribed valacyclovir presented to the emergency department (ED) with a complaint of hallucinations and weakness. This was the patient’s fifth healthcare encounter in three weeks. The first visit was to the ED for heel pain, and he was discharged home after an unremarkable right foot radiograph. The patient then returned to the ED for his second visit with a painful vesicular rash along the second sacral dermatome of his right leg and was prescribed valacyclovir 1 gram orally three times a day for seven days for shingles. Vaccination status was unknown at the time of diagnosis.

On the third ED visit two days later, the patient presented with vomiting after being seen by his primary care doctor that morning. The patient was able to tolerate two doses of valacyclovir; and while being seen by his primary care doctor, his valacyclovir dosing was adjusted to account for his renal disease. The patient also was experiencing hallucinations but was discharged home with the explanation that his symptoms could have been due to dehydration after a “negative workup.” On his fourth visit to the ED seven days later, the patient stated that he would “close his eyes and see bands playing and rolling plains of green grass.” He stated that these images were very vivid but would go away when he opened his eyes. The patient also had difficulty ambulating and generalized weakness. A family member reported that he also had difficulty with finding words.

Vital signs during this fourth ED visit included the following: temperature 99.4° Fahrenheit; pulse 92 beats per minute; respiratory rate 20 respirations per minute; room air pulse oximetry 98%, and a blood pressure of 196/91 millimeters of mercury. Physical examination revealed crusted lesions following the second sacral dermatome on the posterior right leg extending from the sacral region to the lower calf. A neurological exam revealed generalized weakness and difficulty with ambulation without any focal deficits.

Laboratory testing, including complete blood count, metabolic panel and urinalysis were unremarkable except for serum blood urea nitrogen, creatinine and glomerular filtration rate, which were 23.1 milligrams per deciliter (mg/dL) (normal range 6.0–20.0 mg/dL), 3.03 mg/dL (normal range 0.67–1.17 mg/dL) and 22 milliliters per minute (mL/min) (normal is >60 mL/min), respectively. Chest radiograph was unremarkable and brain computed tomography (CT)demonstrated only chronic mild to moderate degenerative changes. Based on the recent diagnosis of shingles, history of immunocompromise and hallucinations with weakness, lumbar puncture was performed. Results included elevated protein with lymphocyte predominance consistent with viral infection. Cerebral spinal fluid (CSF) culture was ordered, and the patient was administered one gram of acyclovir intravenously and admitted to the hospital.

On hospital day one CSF culture demonstrated VZV via polymerase chain reaction (PCR). The patient also underwent brain magnetic resonance imaging (MRI) on hospital day two, which showed moderate chronic microvascular ischemia and abnormal appearance of the distal left vertebral artery. Infectious disease, neurology and hospital medicine teams all evaluated the patient and agreed with the diagnosis of VZV encephalitis in the setting of recent shingles, CSF findings, and patient presentation. The patient was administered a two-week course of acyclovir with improvement of his hallucinations and presenting symptoms prior to discharge on hospital day four.

## DISCUSSION

VZV affects approximately 30% of people in the United States during their lifetime.^1^ Primary infection causes chickenpox or varicella. The virus is never fully eradicated from the body, however, as it travels and lies dormant in the cranial, dorsal root, or autonomic ganglion.^2^ Secondary VZV skin eruption demonstrates a characteristic unilateral, vesicular, and painful eruption that follows a distinct dermatomal distribution. The typical pain pattern of the virus is caused by increased excitability of central nociceptors in the spinal cord causing inflammation and disruption to the nerve cells, making them more sensitive to painful stimuli.^3^

VZV can also cause many different central nervous system (CNS) pathologies if the infection invades the spinal cord or cerebral arteries, including cerebellar ataxia, arteritis, myelitis, meningitis, and encephalitis. CNS infection can occur with primary or secondary reactivation of the virus. Two main risk factors increase the risk for VZV, including age greater than 50 years old and immunocompromise due to reduced T cell-mediated immunity.^4^ Transplant patients are at increased risk compared to the general public with an incidence rate of 17:1000.^5^ The patient in this case study had both of these main risk factors.

VZV encephalitis causes a headache, fever, vomiting, and altered level of consciousness or even seizures. The patient in this case presented with vomiting, mental status changes, and hallucinations. These symptoms can be seen more commonly as side effects due to inappropriately renal-dosed valacyclovir. VZV encephalitis mortality rate for immunocompetent patients is approximately 15% and almost 100% in an immunosuppressed patient, especially if both the liver and lung are infected.^1^,^6^ VZV encephalitis CSF analysis typically demonstrates lymphocytic pleocytosis and elevation of protein both of which occurred in this case. Positive PCR testing in CSF confirms VZV.^7^ CSF anti-VZV antibodies can be performed but cannot be used alone as means for diagnosis of VZV-related neurological conditions.^2^,^8^

**CPC-EM Capsule**

What do we already know about this clinical entity?

*Varicella Zoster Virus (VZV) affects approximately 30% of people in the United States. Encephalitis carries a less favorable prognosis compared to other extra-cutaneous complications of VZV*.

What makes this presentation of disease reportable?

*Emergency physicians are not suspicious of VZV encephalitis as a possible cause in elderly patients with altered mental status. This presentation raises awareness of the importance of considering VZV encephalitis in these patients*.

What is the major learning point?

*Keep a high index of suspicion for VZV encephalitis in the elderly population and those that are immunocompromised, especially with a recent history of rash*.

How might this improve emergency medicine practice?

*Presenting this case and raising awareness amongst emergency physicians can help prevent a delay in diagnosis and improve outcomes*.

Common findings on brain CT specific for VZV encephalitis are a hypodensity in the temporal lobes with possible frontal lobe involvement. The basal ganglia are commonly spared. For MRI, the common findings for VZV encephalitis are edematous changes with hyperdensity in the temporal lobes and inferior frontal lobes with the basal ganglia being spared.^9^,^10^

Treatment of VZV encephalitis is intravenous (IV) acyclovir for seven days in the immunocompetent patient and 10–14 days in the immunosuppressed patient. The patient, in this case, received IV acyclovir for four days and was discharged on two-week course of oral acyclovir. Steroids can be used to reduce inflammation if there is concern for vasculopathy.^11^ The valacyclovir initially prescribed was discontinued by the patient after only two doses due to vomiting. Had the patient taken the full course of medication, neurologic side effects due to renal impairment could have been a cause for his presentation. The incidence of positive PCR CSF in immunosuppressed patients with shingles alone is unknown.

## CONCLUSION

The presence of vomiting, hallucinations, and mental status changes should alert the emergency physician to consider VZV encephalitis, especially in the immunocompromised patient. Prompt lumbar puncture and early administration of IV acyclovir are critical. Also, antiviral medications may cause adverse neurologic effects, especially in older patients with renal disease.
